# Glucagon-like peptide-1 receptor agonists (GLP-1 RAs) may reduce the risk of developing cancer-related lymphedema following axillary lymph node dissection (ALND)

**DOI:** 10.3389/fphar.2024.1457363

**Published:** 2024-09-04

**Authors:** Stav Brown, Audree B. Tadros, Giacomo Montagna, Tajah Bell, Fionnuala Crowley, Emily J. Gallagher, Joseph H. Dayan

**Affiliations:** ^1^ Department of Surgery, Plastic and Reconstructive Surgery Service, Memorial Sloan Kettering Cancer Center, New York, NY, United States; ^2^ Department of Surgery, Breast Service, Memorial Sloan Kettering Cancer Center, New York, NY, United States; ^3^ Division of Hematology and Medical Oncology, Department of Medicine, Icahn School of Medicine at Mount Sinai, New York, NY, United States; ^4^ Brookdale Department of Geriatrics and Palliative Medicine, Icahn School of Medicine at Mount Sinai, New York, NY, United States; ^5^ Department of Medicine, Division of Endocrinology, Diabetes and Bone Diseases, Icahn School of Medicine at Mount Sinai, New York, NY, United States; ^6^ The Institute for Advanced Reconstruction, Plastic and Reconstructive Surgery, Red Bank, Paramus, NJ, United States

**Keywords:** lymphedema, GLP-1R agonists, GLP-1R agonist, GLP-1RAs, cancer-related lymphedema, Ozempic, semaglutide, liraglutide

## Abstract

**Purpose:**

Patients undergoing axillary lymph node dissection (ALND) for breast cancer face a high risk of lymphedema, further increased by high body mass index (BMI) and insulin resistance. GLP-1 receptor agonists (GLP-1RAs) have the potential to reduce these risk factors, but their role in lymphedema has never been investigated. The purpose of this study was to determine if GLP-RAs can reduce the risk of lymphedema in patients undergoing ALND.

**Methods:**

All patients who underwent ALND at a tertiary cancer center between 2010 and 2023 were reviewed. Patients with less than 2 years of follow-up from the time of ALND were excluded. Race, BMI, radiation, chemotherapy history, pre-existing diagnosis of diabetes, lymphedema development after ALND, and the use of GLP-1RAs were analyzed. Multivariate logistic regression analysis was performed to assess if there was a significant reduction in the risk of developing lymphedema after ALND. A sub-group analysis of non-diabetic patients was also performed.

**Results:**

3,830 patients who underwent ALND were included, 76 of which were treated with. GLP-1 RAs. The incidence of lymphedema in the GLP-1 RA cohort was 6.6% (5 patients). Compared to 28.5% (1,071 patients) in the non-GLP-1 RA cohort. On multivariate regression analysis, patients who were treated with GLP-1 RA were 86% less likely to develop lymphedema compared to the non-GLP-1 RA cohort (OR 0.14, 95% CI 0.04–0.32, *p* < 0.0001). A BMI of 25 kg/m 2 or greater was a statistically significant risk factor for developing lymphedema with an odds ratio of 1.34 (95% CI 1.16–1.56, *p* < 0.0001). Diabetes was associated with lymphedema development that closely approached statistical significance (OR 1.32, 95% CI 0.97–1.78, *p* = 0.06). A subgroup analysis solely on non-diabetic patients showed similar results. The odds of developing lymphedema were 84% lower for patients without diabetes treated with GLP1-RAs compared to those who did not receive GLP-1 RAs (OR 0.16, 95% CI 0.05–0.40, *p* < 0.0001).

**Conclusion:**

GLP1-RAs appear to significantly reduce the risk of lymphedema in patientsundergoing ALND. The mechanism of action may be multifactorial and not limited to weight reduction and insulin resistance. Future prospective analysis is warranted to clarify the role of GLP-1RAs in reducing lymphedema risk.

## Introduction

Patients undergoing axillary lymph node dissection (ALND) for breast cancer face a high risk of lymphedema—an incurable and debilitating disease with a reported incidence of 15%–40%. (Cowher et al., 2014; Rockson, 2018; Salinas-Huertas et al., 2021). Severe swelling, life-threatening infections, and lifelong compression are common and degrade the quality of life of millions of breast cancer survivors. Preventing lymphedema is far more preferable to lifelong management of this chronic, intractable, and costly disease. While immediate lymphatic reconstruction has been shown to reduce the risk of lymphedema, it is not widely available and may not always be feasible.

Several factors have been reported to increase the risk of lymphedema following ALND including elevated body mass index (BMI), chemotherapy, and radiation. (Treves, 1957; Mehrara and Greene, 2014; McLaughlin et al., 2017; Helyer et al., 2010a; Petrek et al., 2001; Wilke et al., 2006; Werner et al., 1991; McLaughlin et al., 2008; Rochlin et al., 2023). Weight gain commonly occurs following cancer treatment, exacerbating the risk of lymphedema and its severity in this population. (Gadea et al., 2012; Camoriano et al., 1990; Nichols et al., 2009; Raghavendra et al., 2018). More recent studies have also reported that insulin resistance may increase the risk of lymphedema. (Brown et al., 2023b; Chakraborty et al., 2010; Doruk Analan and Kaya, 2022; Lee et al., 2018; ).

Glucagon-like peptide-1 receptor agonists (GLP-1 RAs) have gained popularity in treating both type 2 diabetes and obesity. (Inzucchi and McGuire, 2008; Jensterle et al., 2022). Weight gain is often a side effect of adjuvant treatment for breast cancer and a risk factor for recurrence, especially in patients with hormone receptor-positive disease. This has resulted in a greater number of breast cancer survivors receiving GLP-1 RAs for weight management. Recently we described the case of a patient treated with GLP-1 RAs for weight gain resulting from their adjuvant breast cancer treatment who also had lymphedema. (Crowley et al., 2024). After GLP-1 RA treatment, we observed a significant improvement in lymphedema including reduced limb volume, improved patient-reported outcome score, and reduced need for compression. (Crowley et al., 2024). However, the potential for reducing the risk of lymphedema using GLP-1 RAs has not yet been explored. The purpose of this study was to determine if GLP-1 RAs can reduce the risk of lymphedema in patients undergoing ALND.

## Methods

**FIGURE 1:**
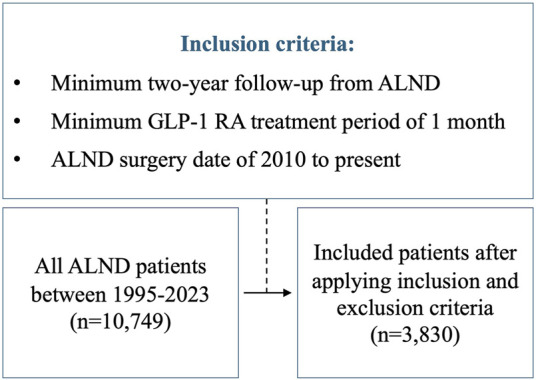
Flow diagram of the patients included in the study.

We reviewed all consecutive ALND cases from 1995 to 2023 at Memorial Sloan Kettering Cancer Center (MSKCC). The study was approved by the Institutional Review Board (IRB #18-177). The assessed independent variables included: GLP-1 RA use, race, preoperative BMI, chemotherapy, radiation, and diabetes. We then applied the following criteria (Figure 1): 1) minimum 2-year follow-up from the time of ALND, 2) Minimum GLP-1 RA treatment period of 1 month, and 3) ALND surgery date of 2010 to present. This last constraint was applied to limit lead-time bias, resulting in a similar mean follow-up time for both the GLP-1 RA group and the non-GLP-1 RA cohort. All patients in the GLP-1 RA group had to have either been on the drug at the time or following ALND. Patients who stopped taking GLP-1 RAs prior to ALND and those diagnosed with lymphedema prior to starting GLP-1 RA treatment were excluded from the study. International classification of diseases (ICD) codes I97.2, I98.0, 457, 457.1 were used to identify patients who developed lymphedema.

### Statistical analysis

Patient demographics and clinical characteristics were summarized using mean and standard deviation (SD) for continuous variables, and percentages for categorical variables. We utilized multivariate logistic regression analysis to evaluate the association between GLP-1 RA use, race, BMI, chemotherapy, radiation, and diabetes on the development of lymphedema. White race was defined as the reference level for the race variable. BMI < 25 was defined as the reference level for the BMI variable. A sub-group analysis of patients without diabetes was also performed. Statistical significance was defined as a *p*-value < 0.05. GraphPad Prism (Dotmatics, Boston, Massachusetts) was used to perform all analyses.

## Results

All patients who underwent ALND at Memorial Sloan Kettering Cancer Center from 1995 to 2023 were evaluated. We identified a total of 10,749 patients, 194 of which were treated with GLP-1RAs. After applying inclusion and exclusion criteria from 2010 to 2023, we identified a total of 3,830 patients who underwent ALND during this time frame, 76 of which were treated with GLP-1 RAs, starting in 2014. Overall demographics and independent variables are listed in Table 1. The overall incidence of lymphedema was 28.0% (1,076 patients). Racial demographics were as follows: 8.4% (n = 322) Asian, 10.4% (n = 399) Black, 73.2% (n = 2,805) White, and 7.9% (n = 304) other. The average BMI was 25.6 ± 5.6 kg/m^2^ and the average age was 53.8 ± 12.8 years. Most patients were female (n = 3,531, 92.2%). 81.7% (n = 3,131) patients received chemotherapy and 64.0% (n = 2,452) of patients received radiation. There were 234 (6.1%) patients with diabetes in the overall dataset. A total of 76 patients (1.9%) were treated with GLP-1 RA. Side effects reported in the GLP-1 RA cohort included nausea (n = 5), diarrhea (n = 4), vomiting (n = 3), and constipation (n = 2). None of the patients on GLP-1 RA in this study had a cancer recurrence.

**TABLE 1:** Demographic and clinical characteristics (n = 3,830).

Variable	Mean (SD) or percentage
Lymphedema	1076 (28.0%)
Race: Asian	322 (8.4%)
Black Other race White	399 (10.4%) 304 (7.9%) 2805 (73.2%)
BMI (kg/m2)	25.6 (5.6)
Chemotherapy	3131 (81.7%)
Radiation	2452 (64.0%)
Treatment with GLP-1RA	76 (1.9%)
Diabetes	234 (6.1%)

Demographic and clinical characteristics of the GLP-1 RA group and the non-GLP-1 RA group are outlined in Table 2. The incidence of lymphedema in the GLP-1 RA cohort was 6.6% (5 patients) compared to 28.5% (1,071 patients) in the non-GLP-1 RA cohort. Both the GLP-1 RA and non-GLP-1 RA groups had similar mean follow-up times from ALND (75.9 ± 37.5 and 75.8 ± 37.9 months, respectively), rates of chemotherapy treatment (81.6% and 81.7%, respectively), and rates of radiation treatment (61.8% and 64.0%, respectively). Racial demographics among the GLP-1 RA and non-GLP-1 RA groups were as follows: Asian (3.9% and 8.5%, respectively), Black (14.5% and 10.3%, respectively), White (73.7% and 73.2%, respectively) and Other (7.9% and 7.9%, respectively). The mean BMI in the GLP-1 RA cohort was 30.7 ± 6.9 kg/m^2^ and in the non-GLP-1 RA cohort was 25.5 ± 5.2 kg/m^2^. The mean age in the GLP-1 RA cohort was 57.3 ± 11.0 years and in the non-GLP-1 RA cohort was 53.7 ± 12.8 years. Most patients were female in both cohorts (GLP-1 RA: n = 69, 90.8%, non-GLP-1 RA: n = 3,462, 92.2%). The incidence of diabetes in the GLP-1 RA cohort was 26.3% (20 patients) and in the non-GLP-1 RA cohort was 6.1% (214 patients). Among those treated with GLP-1 RAs, 47.4% received injectable semaglutide (n = 36), 6.6% were treated with oral semaglutide (n = 5), 25% received dulaglutide (n = 19), 15.7% were treated with liraglutide (n = 12) and 5.3% received exenatide (n = 4).

**TABLE 2:** Demographics and Clinical Characteristics of GLP-1 RA and non- GLP-1RA Cohorts.

Variable	GLP-1RA Group (n = 76)	Non GLP-1RA Group (n = 3754)
Lymphedema	5 (6.6%)	1071 (28.5%)
Race: Asian Black Other race White	3 (3.9%) 11 (14.5%) 6 (7.9%) 56 (73.7%)	319 (8.5%) 388 (10.3%) 298 (7.9%) 2749 (73.2%)
BMI (kg/m2)	30.7 (6.9)	25.5 (5.2)
Chemotherapy	62 (81.6%)	3069 (81.7%)
Radiation	47 (61.8%)	2405 (64.0%)
Diabetes	20 (26.3%)	214 (6.1%)
Follow up (months)	75.9 (37.5)	75.8 (37.9)

n =3830

On multivariate regression analysis (Table 3), patients who were treated with GLP-1 RA were 86% less likely to develop lymphedema compared to the non-GLP-1 RA cohort (OR 0.14, 95% CI 0.04–0.32, *p < 0.0001*). Asian race was a protective factor (OR 0.65*,* 95% CI 0.49–0.86, *p = 0.003*). A BMI of 25 kg/m^2^ or greater was a statistically significant risk factor for developing lymphedema with an odds ratio of 1.34 (95% CI 1.16–1.56, *p < 0.0001*). Both chemotherapy and radiation were associated with an increased risk of developing lymphedema (OR 1.90, 95% CI 1.50–2.41, *p < 0.0001* and OR 2.01, 95% CI 0.97–1.78, *p < 0.0001*, respectively).

**TABLE 3:** Predictive variables for the development of lymphedema (multivariate analysis).

Variable	Odds Ratio	95% CI	*P*-value
GLP-1RA No: Yes:	Ref 0.14	0.04, 0.32	<0.0001*
Race: Asian Black Other race White	0.65 1.06 0.98 Ref	0.49, 0.86 0.84, 1.34 0.75, 1.28	0.003* 0.617 0.882
BMI: <25 kg/m2 ≥25 kg/m2	Ref 1.34	1.16, 1.56	<0.0001*
Chemotherapy No: Yes:	Ref 1.90	1.50, 2.41	<0.0001*
Radiation No: Yes:	Ref 2.01	1.69, 2.39	<0.0001*
Diabetes No: Yes:	Ref 1.32	0.97, 1.78	0.06

GLP-1, RA cohort (n = 76), non-GLP-1, RA cohort (n = 3,754).

*p<0.05.

A subgroup analysis exclusively on patients without diabetes was performed (Table 4). This subgroup, which excluded all patients with diabetes, had similar results as the overall GLP-1 RA group using multivariate regression. The odds of developing lymphedema were 84% lower for patients without diabetes treated with GLP1-RAs compared to those who did not receive GLP-1 RAs (OR 0.16, 95% CI 0.05–0.40, *p < 0.0001*). The incidence of lymphedema in the GLP-1 RA group without diabetes was 7.1% (n = 4), and in the non-GLP-1 RA treated group without diabetes was 28.2% (n = 997) with a mean follow-up period of 66.6 ± 33.0 and 75.0 ± 37.2 months, respectively.

**TABLE 4:** Predictive variables for the development of lymphedema excluding diabetic patients (multivariate analysis).

Variable	Odds ratio	95% CI	*P*-value
GLP-1RA No: Yes:	Ref 0.16	0.05, 0.40	0.0006*
Asian	0.60	0.44, 0.80	0.0009*
Black	1.04	0.81, 1.32	0.767
Other race White	0.98 Ref	0.74, 1.28	0.904
BMI: <25 kg/m^2^ ≥25 kg/m^2^	Ref 1.34	1.15, 1.56	<0.0002*
Chemotherapy No: Yes:	Ref 1.91	1.50, 2.45	<0.0001*
Radiation No: Yes:	Ref 1.99	1.66, 2.38	<0.0001*

GLP-1, RA cohort (n = 56), non-GLP-1, RA cohort (n = 3,541).

*p<0.05.

## Discussion

Patients undergoing ALND for breast cancer face both a high risk of lymphedema as well as a high risk of weight gain after breast cancer diagnosis. The results in this study demonstrated a 28% overall incidence of lymphedema after ALND. This rate likely underestimates the true incidence of lymphedema in this cohort given that the diagnosis was based solely on diagnosis codes, which may not capture all cases. Nonetheless, this figure is consistent with the published literature citing a lymphedema incidence of approximately 1 in 3 patients or higher, depending on how lymphedema is defined (Cowher et al., 2014; Rockson, 2018; Salinas-Huertas et al., 2021). Several publications, including a large study by MD Anderson Cancer Center, reported significant weight gain in 33.7% of breast cancer survivors with hormone receptor (HR)-positive and human epidermal growth factor receptor 2 (HER2)-negative tumors (Gadea et al., 2012; Camoriano et al., 1990; Nichols et al., 2009; Raghavendra et al., 2018). Elevated BMI has been reported to be a significant risk factor for developing lymphedema after ALND (Treves, 1957; McLaughlin et al., 2017; Helyer et al., 2010a; Petrek et al., 2001; McLaughlin et al., 2008; Rochlin et al., 2023). Finally, insulin resistance may impair lymphatic function and increase the risk of lymphedema (Chakraborty et al., 2010; Doruk Analan and Kaya, 2022; Lee et al., 2018).

Currently, there is no FDA-approved drug indicated for the prevention or treatment of lymphedema—a tremendous unmet need for such a prevalent, debilitating and incurable condition. While there have been a variety of publications on off-label and investigational agents used to treat lymphedema, none have gained widespread acceptance. These treatments were each targeting various components of the underlying pathophysiology of lymphedema. While removing axillary lymph nodes does interrupt lymphatic flow, it is the dysregulated immune response that results in chronic lymphedema (Brown et al., 2023a). The injury from ALND causes lymph stagnation, inducing a T-cell-mediated inflammatory cascade. This leads to scarring, smooth muscle dysfunction, and increased permeability of the lymphatic vessels. An amplified cycle of lymph accumulation and inflammation ensues. Progressive fibrosis and localized fat accumulation follow (Brown et al., 2023a). Pharmacologic treatment strategies range from inducing lymphangiogenesis to blocking the various targets implicated in the inflammatory and fibrotic pathways (Li et al., 2020; Szuba et al., 2002; Cheung et al., 2006; Jin et al., 2009; Hadamitzky et al., 2016; Nakamura et al., 2009; Tian et al., 2017; Rockson et al., 2018; Ubenimex in Adult Patients with, 2021; Cribb et al., 2021; Gardenier et al., 2017; U.S. Prescribing information; García Nores et al., 2018; Zampell et al., 2012; Avraham et al., 2013; Mehrara et al., 2021; Debrah et al., 2006; Mand et al., 2012; Furlong-Silva et al., 2021; Brown et al., 2022a; Avraham et al., 2010; Sano et al., 2020; Clavin et al., 2008; Yoon et al., 2020; Brown et al., 2023c). However, most of these studies have a small sample size and limited follow-up making efficacy difficult to interpret. Currently there is no widely used agent for lymphedema treatment or prevention.

Axillary reverse mapping is a surgical technique that can be employed during sentinel lymph node biopsy or axillary dissection to reduce the risk of lymphedema (Dayan et al., 2015; Boneti et al., 2009; Tummel et al., 2017). Different agents are injected into the breast and upper extremity allowing the surgeon to identify which lymph nodes drain the upper limb so they can be avoided if possible. However, some lymph nodes share drainage from both the upper limb and the breast and still need to be removed. This technique is also not widely available.

Immediate lymphatic reconstruction using microsurgical technique has been reported to reduce the risk of lymphedema. At the time of ALND, a lymphovenous bypass (LVB) can be performed between the divided lymphatics and an adjacent vein in order to restore lymphatic flow out of the upper limb (Johnson et al., 2021; Johnson and Singhal, 2018; Coriddi et al., 2023; Boccardo et al., 2009). Alternatively, in more radical axillary exenterations, lymphatic tissue replacement using a vascularized omentum free flap has been reported to significantly reduce both the risk of lymphedema as well as painful axillary contracture (Brown et al., 2024). Vascularized lymph node transplant (VLNT) has shown promise in treating an already established disease, improving limb volumes, incidence of cellulitis, and quality of life (Losco et al., 2022; Francis et al., 2022; Agko et al., 2018; Akita et al., 2015; Beederman et al., 2020; Bolletta et al., 2022; Brown et al., 2022b; Chang et al., 2020; Cheng et al., 2013; Coroneos et al., 2022; Di Taranto et al., 2021; Dionyssiou et al., 2016; Garza et al., 2022; Gratzon et al., 2017; Lin et al., 2020; Maldonado et al., 2017; Patel et al., 2015; Schaverien et al., 2021; Schaverien et al., 2022). However, these procedures are also not widely available, require microsurgical skill, and may not be technically feasible in every patient. All these surgical modalities focus on the local lymphatic injury but do not address the additional risk factors of elevated BMI or insulin resistance. A holistic approach of optimizing all risk factors in addition to the axillary dissection itself may provide further risk reduction. If anything can be done to significantly reduce risk, it would be a welcome advance in the effort to avoid the permanent morbidity of lymphedema.

GLP-1RAs have emerged as highly effective treatments for individuals with type 2 diabetes and obesity. (Inzucchi and McGuire, 2008; FDA FaDA, 2020) The most commonly used GLP-1 RA in this study was semaglutide. This drug is currently indicated for glycemic control in people with type 2 diabetes and for weight management in patients with a BMI ≥ 30 kg/m^2^ or patients with a BMI ≥ 27 kg/m^2^ with at least one weight-related comorbidity such as hypertension, type 2 diabetes, or hypercholesterolemia (Jensterle et al., 2022).

The purpose of this study was to examine if patients who were being treated with GLP-1 RAs after ALND had a reduced risk of developing lymphedema. We found a significant risk reduction for lymphedema in both patients with and without diabetes alike. While there are many inherent limitations in any retrospective study, the risk reduction was found to be dramatic enough to warrant future prospective investigation. If efficacy is demonstrated in a future prospective study, GLP-1 RAs could represent the first medical therapy to reduce the risk of lymphedema in the many women who face this incurable prospect. Avoiding lymphedema is far preferable to treating it, as there is currently no known cure, and it requires lifelong management. Preventative therapies in lymphedema will not only preserve quality of life but also significantly reduce the cost burden on the patient and the healthcare system at large (Boyages et al., 2017; Roberson et al., 2021). By highlighting the potential benefits of GLP-1RAs, the current study opens new avenues for preventing and managing lymphedema in cancer-survivors.

**FIGURE 2:**
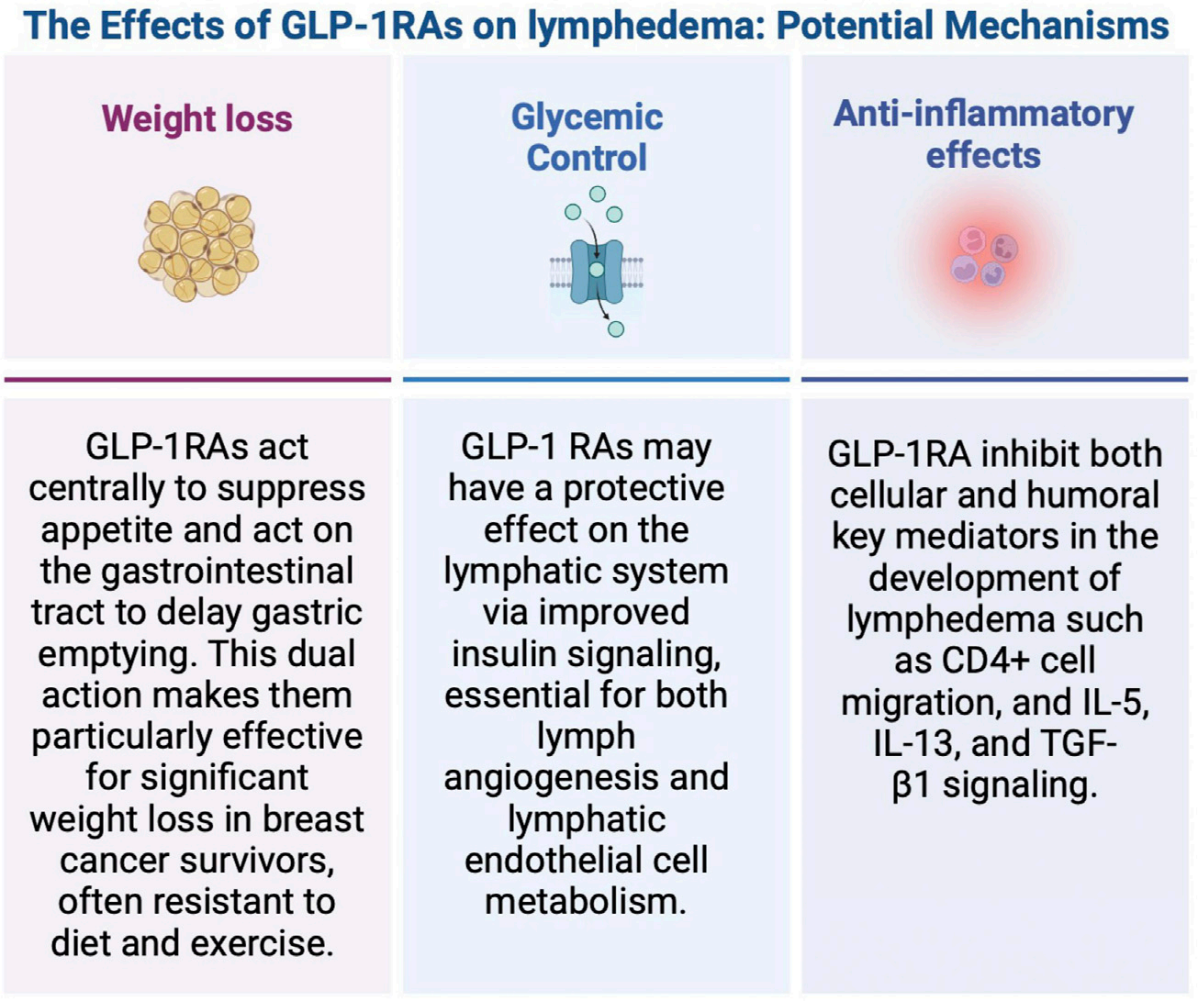
The Effects of GLP-1RAs on lymphedema: potential mechanisms. Created with Biorender.

The next logical question is how do GLP-1 RAs reduce the risk of lymphedema? (Figure 2). Regarding weight loss, GLP-1RAs act centrally to suppress appetite and act on the gastrointestinal tract to delay gastric emptying (Zhao et al., 2021; Shah and Vella, 2014). As high BMI has been reportedly associated with greater lymphedema risk, it seems that GLP-1 RAs could potentially reduce lymphedema risk with weight loss. McLaughlin et al. conducted a prospective study in 936 women with breast cancer over a period of 5 years who underwent SLNB alone or ALND (McLaughlin et al., 2008). Preoperative weight, current weight, preoperative BMI and current BMI (*p* < 0.0001) were independent risk factors for the development of lymphedema. Helyer et al. conducted prospective arm measurements of 137 breast cancer patients every 6 months from date of diagnosis for 2 years (Helyer et al., 2010b). Patients with a preoperative BMI >30 had an odds ratio of 2.93 (95% CI 1.03–8.31) compared with those with a BMI of <25 of developing lymphedema. In our study using multivariate regression analysis, we found patients with a BMI ≥ 25 were 1.3 times more likely to develop lymphedema after ALND (OR = 1.3, *P* < 0.0001) compared to patients with a BMI < 25. These findings demonstrate BMI is an independent risk factor for developing lymphedema, and not limited to patients with a BMI >30. However, there are some important points to highlight specific to this database which may affect the true odds ratio. The BMI recorded in this database is a single preoperative BMI and does not represent the BMI at the postoperative time point. Consequently, it does not account for weight gain that would be expected in some patients receiving adjuvant therapy, nor does it account for any expected weight loss as a result of GLP-1 RA use. These presumed changes in BMI *over time* are not accounted for in this dataset but would likely increase the odds ratio of the effect of BMI on lymphedema risk based on what has been previously published.

While high BMI is a known risk factor for lymphedema and weight gain is also associated with increased risk, the impact of weight loss on lymphedema has yet to be fully explored. Two publications by Shaw et al. concluded weight loss led to a significant reduction in lymphedema (Shaw et al., 2007a; Shaw et al., 2007b). In contrast, two studies by Schmitz et al. observed no improvement in lymphedema with weight loss. However, the mean weight reduction in these last two studies using diet and exercise was mild and ranged from 3.2% to 8% (Schmitz et al., 2013; Schmitz et al., 2019). GLP-1 RAs, in contrast have reported a weight reduction of up to 20% of the patient’s body weight (Jensterle et al., 2022; Jensen et al., 2023; Kristensen et al., 2019; Jastreboff et al., 2022). We recently published a case report with objective multimodal evidence of resolution of lymphedema with GLP-1 RA use leading to a 24% loss in body weight (Crowley et al., 2024). In conclusion, the degree of weight loss probably makes a significant difference, whereas modest levels of weight loss are less likely to matter.

Is there any evidence that weight loss can reduce the risk of lymphedema? In a study by Roberts et al. (2021), the findings suggested that weight loss did not reduce lymphedema risk. However, the average weight loss was only 8 pounds (3.6 kg) in 49 months—a modest reduction over a long time—and likely inadequate to make a positive impact in lymphedema. In contrast, semaglutide, is associated with a mean weight reduction of 28 pounds in 17 months versus placebo (Haider and Lipska, 2022). GLP-1 RAs provide a powerful means for significant weight loss, whereas for most breast cancer survivors, diet and exercise have typically modest results.

In addition to weight loss, GLP-1RAs may affect lymphedema, given their beneficial effects on glucose control. In this study, diabetes was associated with lymphedema development that closely approached statistical significance (*p = 0.06*). Chronic hyperglycemia has been shown to lead to lymphatic dysfunction, and insulin resistance may be a risk factor for developing lymphedema (Chakraborty et al., 2010; Doruk Analan and Kaya, 2022; Lee et al., 2018). Insulin resistance has been shown to compromise both the integrity and function of lymphatic endothelial cells (Lee et al., 2018). Consequently, GLP-1 RAs may have a protective effect on the lymphatic system but this is an area of future investigation.

In addition to weight loss and glucose control, GLP-1 RAs affect both immunologic and inflammatory pathways which may have a direct and positive effect on lymphatic function (Park et al., 2024; Alharbi, 2024; Bendotti et al., 2022/08; Farr et al., 2016; Chen et al., 2022; Mehdi et al., 2023). The pathophysiology of lymphedema is complex and includes features of a dysregulated immune response leading to chronic inflammation, lymphatic dysfunction, and fibrofatty proliferation. It has been established that activated CD4^+^ T cells and differentiation into T-helper 2 (Th2) cells are at the front end of this process (Avraham et al., 2013; Ly et al., 2019). Downstream production of pro-inflammatory cytokines such as interleukin-4 (IL-4), interleukin-5 (IL-5), and interleukin-13 (IL-13) contribute to chronic inflammation and fibrosis (Brown et al., 2023a). GLP-1 RAs have been shown to inhibit chemokine-related migration of human CD4^+^ lymphocytes (Liberman et al., 2013). Additionally, GLP-1RAs have demonstrated efficacy in reducing allergic responses in asthma by inhibiting the activation of the NF-kB pathway. This inhibition leads to decreased numbers and reduced migration of CD4^+^ T cells, as well as a reduction in the release of IL-5 and IL-13 (Toki et al., 2021; Wu and Peebles, 2021). Liraglutide was also shown to mitigate fibrosis in mice through the downregulation of NF-kB signaling and the decrease in levels of TGF-β1, a key regulator of fibrosis in cancer-related lymphedema (Gou et al., 2014; Baik et al., 2022; Kataru et al., 2019). These anti-inflammatory and immunologic effects of GLP-1 RAs are of interest as weight loss alone may not be the only therapeutic mechanism as it relates to lymphedema.

Safety is always of utmost concern—an important area of ongoing research is determining the effect of GLP-1 RAs on cancer risk and recurrence. A recent retrospective study involving a large sample size of 1,890,020 diabetic patients found that GLP-1RAs were associated with a significantly lower risk of hepatocellular carcinoma compared to insulin and other diabetic medications over a 5-year follow-up period (Wang et al., 2024a). Similarly, a nationwide retrospective cohort study of 1,221,218 patients with diabetes demonstrated a reduced risk of colorectal cancer with GLP-1RA use over a 15-year follow-up period (Wang et al., 2024b). Another retrospective cohort study, which included 543,595 diabetic patients, found no increased risk of pancreatic cancer associated with GLP-1RA use compared to basal insulin over a 9-year follow-up period (Dankner et al., 2024). A more recent meta-analysis including 37 randomized controlled trials did not find an increase in the risk of any type of cancer on GLP-1 RAs (Nagendra et al., 2023).

Pre-clinical studies investigating the impact of GLP-1 RAs specifically on breast cancer progression have produced conflicting results, which vary based on the receptor status of the cancer cell lines studied (Ligumsky et al., 2012; Alanteet et al., 2024; Alanteet et al., 2021; Zhao et al., 2018; Shadboorestan et al., 2021; Liu et al., 2022). Ligumsky et al. demonstrated that GLP-1 and its analog exendin-4 enhance the apoptosis of estrogen receptor-positive breast cancer cells *in vitro* (Ligumsky et al., 2012). In an *in vitro* study by Zhao et al., liraglutide reduced cell proliferation and increased cell apoptosis in estrogen receptor-positive breast cancer cells (Zhao et al., 2018). Two recent *in vitro* studies by Alanteet et al. supported liraglutide’s anti-proliferative and pro-apoptotic effects in cancer cells (Alanteet et al., 2024; Alanteet et al., 2021). In contrast to liraglutide’s effects in estrogen receptor-positive breast cancer, a recent *in vitro* study observed that liraglutide stimulates the growth of triple-negative breast cancer cells (Shadboorestan et al., 2021). Additionally, Liu et al. (2022) found that liraglutide increased GLP-1 receptor expression in triple negative breast cancer cells and tumors in a mouse model. Further studies are needed to better clarify any positive or negative effects on breast cancer.

### Limitations

There are inherent limitations in any registry-oriented retrospective analysis. Using a large sample size, we made every attempt to mitigate the effect of confounding factors. Only patients with a minimum follow-up period of 2 years were included because the majority of lymphedema following ALND will occur during this period (Montagna et al., 2022; Norman et al., 2009; Ren et al., 2022). In a 5 year prospective study by Norman, et al., 80% of patients developed lymphedema within the first 2 years of surgery (Norman et al., 2009). Additionally, we ensured that both the non-GLP-1 RA and GLP-1 RA treatment cohorts had similar mean follow-up times (75.9 months, SD = 37.5 and 75.8 months, SD = 37.9, respectively) to control for lead-time bias. Finally, a subgroup analysis of non-diabetic patients was performed to exclude confounding factors in our diabetic population. We performed this subgroup analysis for two reasons: 1) to eliminate the potentially confounding factor of using other diabetic medications such as metformin or insulin, and 2) we did not have data on the severity of diabetes such as hemoglobin A1c which may be an additional confounding factor. However, there are limitations specific to this study in addition to its retrospective nature and relatively small proportion of patients treated with GLP-1 RAs. Lymphedema was defined by the presence of a diagnosis code in the registry without an objective metric. Data on the severity of lymphedema was also not available. For chronic conditions like type 2 diabetes, GLP-1 RAs are often intended for long-term use. However, the duration may be adjusted based on the patient’s response to treatment, side effects, combination with other antidiabetic medications, and overall health goals. For semaglutide, for example, the initial dose is typically 0.25 mg once a week for the first 4 weeks. After the initial period, the dose may be increased to 0.5 mg up to a maximum of 2 mg once a week if additional glycemic control is needed. The maintenance dose is usually adjusted to each patient’s response, diet, exercise regimen, and tolerance. (Available at) Therefore, the dose, total duration, and type of GLP-1 RA used were variable among the cohort studied. These limitations alongside the small proportion of patients treated with GLP-1RAs underscores the importance of conducting a prospective trial with predetermined dosing regimens to accurately assess the impact of GLP-1 RAs on lymphedema and to guide future treatment protocols.

## Conclusions

Taking into account the limitations of this study along with the large sample size and constraints, there was a still a significant 86% risk reduction in lymphedema in the GLP-1 RA group. At minimum, these findings warrant future prospective study given the significant implications. Lymphedema risk reduction has largely focused on addressing the surgical defect, but a more holistic approach may be appropriate. GLP-1 RAs can reliably treat a known risk factor for developing lymphedema—elevated BMI. Many women with breast cancer experience dramatic weight gain resistant to diet and exercise. Future prospective studies are needed to assess the role of GLP-1RAs relating to lymphedema and further understand the mechanism of action and safety. The study of GLP-1 in patients at risk for developing lymphedema as well as those who have lymphedema is a worthwhile pursuit. In most cases, women undergo an axillary dissection dreading the prospect of lymphedema with little to do but hope for the best. If there is any way to reduce the risk of this incurable disease it would a welcome benefit to those facing a breast cancer diagnosis.

## Data Availability

The raw data supporting the conclusions of this article will be made available by the authors, without undue reservation.
